# Supplementary material for: Translation, reliability, and validity of Amharic versions of the Pelvic Floor Distress Inventory (PFDI-20) and Pelvic Floor Impact Questionnaire (PFIQ-7)
Source: PLoS One. 2022 Nov 17;17(11):e0270434. doi: 10.1371/journal.pone.0270434 (PMC9671332; doi:10.1371/journal.pone.0270434)
Supplement: S1 File — (PDF) [file pone.0270434.s001.pdf]

በማህፀን ድጋፍ መጎዳት ምክንያት የሚመጡ የሽንት ወይም ሰገራ አለመቆጣጠር እና የማህጸን ወደ ውጭ መዉጣት ችግሮች የሚያሳያቸውን ምልክቶች መጠየቂያ ቅጽ (PFDI-20)

መመሪያ: እባክዎን በሚከተለው የዳሰሳ ጥናት ውስጥ ያሉትን ሁሉንም ጥያቄዎች ይመልሱ። እነዚህ ጥያቄዎች የተወሰኑ የአንጀት፣ የፊኛ ወይም የዳሌ ምልክቶች ካለብዎት እና፣ ካለብዎት ደግሞ፣ ምን ያህል እንደሚያስቸግሯችሁ ይጠይቁዎታል። በተገቢው ሳጥን ወይም ሳጥኖች ውስጥ x በማስቀመጥ ይመልሱ። ለእነዚህ ጥያቄዎች መልስ በሚሰጡበት ጊዜ፣ እባክዎን **የባለፉትን 3 ወራት** ግምት ውስጥ ያስገቡ።

ይህ መጠየቂያ ቅጽ 20 ጥያቄዎችንና ሶስት ራሳቸውን የቻሉ መሳሪያዎችን የያዘ ነው። ሁሉም ጥያቄዎች አንድ አይነት የአመላለስ ስልት ሲኖራቸው፤ ምላሻቸውም ከዜሮ እስከ አራት ነው።

_________________ይህ ምልክት አለብዎት? አለኝ ☐ የለኝም ☐

ካለብዎት ምንያህል ያስጨንቀዎታል? 1 ☐ በፍጹም 2 ☐ መጠነኛ 3 ☐ መካከለኛ 4 ☐ ከፍተኛ

ባለፈው ሶስት ወር ውስጥ የሚከተሉትን ምልክቶች በምን ያህል አይተሻል?					
**ከማህፀን መውረድና ወደ ውጭ መውጣት ጋር የተያያዙ ምልክቶች** (POPDI-6)		በፍጹም	መጠነኛ	መካከለኛ	ከፍተኛ
1	በታችኛው የሆድሽ ክፍል የመጫን ስሜት ይሰማሻል?	1	2	3	4
2	በዳሌሽ ወይም ወገብሽ አካባቢ የመክበድ ስሜት ይሰማሻል?	1	2	3	4
3	በማህጸንሽ አካባቢ በአይን የሚታይ ወይም የሚዳሰስና ወደ ዉጭ የወጣ ወይም የተወተፈ ነገር አለሽ?	1	2	3	4
4	እዳሪ ለመውጣት ወይም ለመጸዳዳት የብልትሽን ወይም የፊንጢጣሽን አካባቢ መደገፍ ይጠብቅብሻል?	1	2	3	4
5	ሽንትሽን ከሸናሽ በኋላ ሙሉ ለሙሉ ሽንተሽ ያልጨረስሽ መስሎ ይሰማሻል?	1	2	3	4
6	ሽንት ለመሽናት ወይም ሙሉ ለሙሉ ሽንተሽ ለመጨረስ በብልትሽን አካባቢ የወጣውን ነገር በእጅሽ ወደላይ መግፋት ይጠብቅብሻል?	1	2	3	4
**ከአይነ-ምድር መውጫ ጋር የተያያዙ ምልክቶች** (CRAD-8)					
7	ሰገራሽን/አይነ-ምድርሽን ለመጸዳዳት በሀይል ማማጥ ይጠብቅብሻል?	1	2	3	4
8	ሰገራሽን/አይነ-ምድርሽን ከተጸዳዳሽ በኋላ ሙሉ ለሙሉ ሰገራሽን ያልጨረስሽ መስሎ ይሰማሻል?	1	2	3	4
9	የቀጠነ ሰገራ/እዳሪ ሳይኖርሽ የማምለጥ ወይም ያለመቆጣጠር ችግር አለብሽ?	1	2	3	4
10	የቀጠነ ሰገራ/እዳሪ የማምለጥ ወይም ያለመቆጣጠር ችግር አለብሽ?	1	2	3	4
11	ፈስ ወይም ጋዝ ያመልጥሻል?	1	2	3	4
12	ሰገራሽን/አይነ-ምድርሽን ስትጸዳጅ ህመም/ስቃይ ይሰማሻል?	1	2	3	4
13	ሰገራሽን/አይነ-ምድርሽን ለመጸዳዳት ወደ መጸዳጃ ቤት ለመሄድ የመጣደፍ ስሜት ይሰማሻል?	1	2	3	4
14	በምትጸዳጅበት ወይም በኋላ በፊንጢጣሽ በኩል የሚወተፈ ወይም የሚወጣ ነገር አለሽ?	1	2	3	4
**ከሽንት ጋር የተያያዙ ምልክቶች** (UDI-6)					
15	ሽንት ቶሎቶሎ ይመጣሻል?	1	2	3	4
16	ሽንት ማጣደፍ ሲኖርሽ የሚሸናበት ቦታ ሳትደርሽ ሽንት ያመልጥሻል?	1	2	3	4
17	ስትስቂ፡ ስታነጥሽ ወይንም ስትስይ ሽንት ያመልጥሻል?	1	2	3	4
18	ሽንትሽ እየተንጠበጠበ ያስቸግርሻል?	1	2	3	4
19	ሽንትሽን ለመሽናት ትቸገሪያለሽ?	1	2	3	4
20	ከእንብርትሽ በታች ወይም በማህጸንሽ አካባቢ ህመም አለሽ?	1	2	3	4

በማህፀን ደጋፊ መጎዳት ምክንያት የሚመጡት የሽንት ወይም ሰገራ አለመቆጣጠር እና የማህጸን ወደ ውጭ መዉጣት ችግሮች በህሙማኑ ላይ የሚያሳድሩት ተጽኖዎች መጠየቂያ ቅጽ (PFIQ-7)

መመሪያ፡ አንዳንድ በህመሙ የተያዙ ሴቶች የፊኛ፣ የአንጀት ወይም የሴት ብልት ምልክቶች በእንቅስቃሴዎቻቸው፣ በግንኙነታቸው እና በስሜታቸው ላይ ተጽዕኖ እንደሚያሳድሩ ይገነዘባሉ። ለእያንዳንዱ ጥያቄ **ባለፉት 3 ወራት ውስጥ** እንቅስቃሴዎችዎ፣ ግንኙነቶችዎ ወይም ስሜቶችዎ በፊኛዎ፣ በአንጀትዎ ወይም በብልትዎ ምልክቶች ወይም ሁኔታዎች ምን ያህል እንደተጎዱ በተሻለ ሁኔታ **የሚገልጽውን ቁጥር በማክበብ** በምላሹ ላይ ያስቀምጡ። እባክዎን ለእያንዳንዱ ጥያቄ በሁሉም 3 አምዶች ውስጥ መልስ እንዳለዎት ያረጋግጡ።

	እንዴት ነው የችግሩ/ህመሙ ምልከቶች በእነዚህ ክፍሎች ⇒ ⇒ ⇒ ⇒<br>በህይዎትሽ ላይ በምን ያህል ተጽኖ ፈጠሩ? ⇓	ሽንት-ፊኛ/ሽንት	አንጀት/ፊንጢጣ	ማህጸን/ብልት/ዳሌ
1	የቤት ውሰጥ ስራዎችሽ ላይ ለምሳሌ ልብስ አጠባ/ማጠብ፤ ምግብ ማብሰል፤ ቤት ማጽዳት፤ እህል መፍጨት	0. በፍፁም<br>1. በመጠኑ<br>2. መካከለኛ<br>3. ከፍተኛ	0. በፍፁም<br>1. በመጠኑ<br>2. መካከለኛ<br>3. ከፍተኛ	0. በፍፁም<br>1. በመጠኑ<br>2. መካከለኛ<br>3. ከፍተኛ
2	የእለት-እለት እንቅስቁሴሽ ላይ ለምሳሌ ውሀ ከወንዝ መቅዳት፤ ገቢያ ሄዶ እህል ማቅናት/መሸመት፤ እርሻ ማሳ መሄድና ማገዝ፤ እንጨት መልቀም	0. በፍፁም<br>1. በመጠኑ<br>2. መካከለኛ<br>3. ከፍተኛ	0. በፍፁም<br>1. በመጠኑ<br>2. መካከለኛ<br>3. ከፍተኛ	0. በፍፁም<br>1. በመጠኑ<br>2. መካከለኛ<br>3. ከፍተኛ
3	እንደ ፊልም ወይም የሙዚቃ ትርኢት፤ጭፈራ፤እስክስታ የመሳሰሉ መዝናኛዎች ላይ	0. በፍፁም<br>1. በመጠኑ<br>2. መካከለኛ<br>3. ከፍተኛ	0. በፍፁም<br>1. በመጠኑ<br>2. መካከለኛ<br>3. ከፍተኛ	0. በፍፁም<br>1. በመጠኑ<br>2. መካከለኛ<br>3. ከፍተኛ
4	ከቤት ከ30 ደቂቃዎች በላይ ለሆነ እርቀት በመኪና ወይም በአውቶቢስ የመጓዝ ሁኔታሽ ላይ	0. በፍፁም<br>1. በመጠኑ<br>2. መካከለኛ<br>3. ከፍተኛ	0. በፍፁም<br>1. በመጠኑ<br>2. መካከለኛ<br>3. ከፍተኛ	0. በፍፁም<br>1. በመጠኑ<br>2. መካከለኛ<br>3. ከፍተኛ
5	ከቤት ውጭ ባሉሽ ማህበራዊ ህይወት ወይም ስራ ላይ፡ ለምሳሌ ቤተክርስቲያን/መስጊድ መሄድ፤ ቀብር መድረስ ፤ዘመዶችሽን/ጓደኞችሽን/ቤተሰቦችሽን መጠየቅ፤ ሰርግ፤ ማህበር/ጽዋ፤ ሰንበቴ....	0. በፍፁም<br>1. በመጠኑ<br>2. መካከለኛ<br>3. ከፍተኛ	0. በፍፁም<br>1. በመጠኑ<br>2. መካከለኛ<br>3. ከፍተኛ	0. በፍፁም<br>1. በመጠኑ<br>2. መካከለኛ<br>3. ከፍተኛ
6	ስሜትሽ ላይ፡ ለምሳሌ መደበት፤ ማዘን፤ መስጋት	0. በፍፁም<br>1. በመጠኑ<br>2. መካከለኛ<br>3. ከፍተኛ	0. በፍፁም<br>1. በመጠኑ<br>2. መካከለኛ<br>3. ከፍተኛ	0. በፍፁም<br>1. በመጠኑ<br>2. መካከለኛ<br>3. ከፍተኛ
7	ተስፋ የማጣት ስሜት ላይ	0. በፍፁም<br>1. በመጠኑ<br>2. መካከለኛ<br>3. ከፍተኛ	0. በፍፁም<br>1. በመጠኑ<br>2. መካከለኛ<br>3. ከፍተኛ	0. በፍፁም<br>1. በመጠኑ<br>2. መካከለኛ<br>3. ከፍተኛ
