# Supplementary material for: Translation, reliability, and validity of Amharic versions of the Pelvic Floor Distress Inventory (PFDI-20) and Pelvic Floor Impact Questionnaire (PFIQ-7)
Source: PLoS One. 2022 Nov 17;17(11):e0270434. doi: 10.1371/journal.pone.0270434 (PMC9671332; doi:10.1371/journal.pone.0270434)
Supplement: S2 File — (PDF) [file pone.0270434.s002.pdf]

English version of the Short form of PFDI-20 and PFIQ-7

Pelvic Floor Distress Inventory–short form 20

Instructions: Please answer all of the questions in the following survey. These questions will ask you if you have certain bowel, bladder, or pelvic symptoms and, if you do, how much they bother you. Answer these by putting an **X** in the appropriate box or boxes. While answering these questions, please consider your symptoms over the **last 3 months.**

The PFDI-20 has 20 items and 3 scales.
All items use the following format with a response scale from 0 to 4.

Do you _____________________?

☐ No; ☐ Yes
0

If yes, how much does it bother you?
☐ 1 ☐ 2 ☐ 3 ☐ 4
Not at all Somewhat Moderately Quite a bit

Scales
Pelvic Organ Prolapse Distress Inventory 6 (POPDI-6):

1. Usually experience *pressure* in the lower abdomen?
2. Usually experience *heaviness or dullness* in the pelvic area?
3. Usually have a bulge or something falling out that you can see or feel in your vaginal area?
4. Ever have to push on the vagina or around the rectum to have or complete a bowel movement?
5. Usually experience a feeling of incomplete bladder emptying?
6. Ever have to push up on a bulge in the vaginal area with your fingers to start or complete urination?

Colorectal-Anal Distress Inventory 8 (CRADI-8):

7. Feel you need to strain too hard to have a bowel movement?
8. Feel you have not completely emptied your bowels at the end of a bowel movement?
9. Usually lose stool beyond your control if your stool is well formed?
10. Usually lose stool beyond your control if your stool is loose?
11. Usually lose gas from the rectum beyond your control?
12. Usually have pain when you pass your stool?
13. Experience a strong sense of urgency and have to rush to the bathroom to have a bowel movement?
14. Does part of your bowel ever pass through the rectum and bulge outside during or after a bowel movement?

Urinary Distress Inventory 6 (UDI-6):

15. Usually experience frequent urination?
16. Usually experience urine leakage associated with a feeling of urgency, that is, a strong sensation of needing to go to the bathroom?
17. Usually experience urine leakage related to coughing, sneezing, or laughing?
18. Usually experience small amounts of urine leakage (that is, drops)?
19. Usually experience difficulty emptying your bladder?
20. Usually experience *pain* or *discomfort* in the lower abdomen or genital region?

Scale scores: Obtain the mean value of all of the answered items within the corresponding scale (possible value 0 to 4) and then multiply by 25 to obtain the scale score (range 0 to 100). Missing items are dealt with by using the mean from answered items only.

PFDI –20 Summary Score: Add the scores from the 3 scales together to obtain the summary score (range 0 to 300).

Pelvic Floor Impact Questionnaire–short form 7

Instructions: Some women find that bladder, bowel, or vaginal symptoms affect their activities, relationships, and feelings. For each question place an **X** in the response that best describes how much your activities, relationships, or feelings have been affected by your bladder, bowel, or vaginal symptoms or conditions **over the last 3 months**. Please make sure you mare an answer in **all 3 columns** for each question.

How do symptoms or conditions relate to the following →→→→ usually affect your ↓	***Bladder or urine***	***Bowel or rectum***	***Vagina or pelvis***
1. Ability to do household chores (cooking, housecleaning, laundry)?	☐ Not at all<br>☐ Somewhat<br>☐ Moderately<br>☐ Quite a bit	☐ Not at all<br>☐ Somewhat<br>☐ Moderately<br>☐ Quite a bit	☐ Not at all<br>☐ Somewhat<br>☐ Moderately<br>☐ Quite a bit
2. Ability to do physical activities such as walking, swimming, or other exercise?	☐ Not at all<br>☐ Somewhat<br>☐ Moderately<br>☐ Quite a bit	☐ Not at all<br>☐ Somewhat<br>☐ Moderately<br>☐ Quite a bit	☐ Not at all<br>☐ Somewhat<br>☐ Moderately<br>☐ Quite a bit
3. Entertainment activities such as going to a movie or concert?	☐ Not at all<br>☐ Somewhat<br>☐ Moderately<br>☐ Quite a bit	☐ Not at all<br>☐ Somewhat<br>☐ Moderately<br>☐ Quite a bit	☐ Not at all<br>☐ Somewhat<br>☐ Moderately<br>☐ Quite a bit
4. Ability to travel by car or bus for a distance greater than 30 minutes away from home?	☐ Not at all<br>☐ Somewhat<br>☐ Moderately<br>☐ Quite a bit	☐ Not at all<br>☐ Somewhat<br>☐ Moderately<br>☐ Quite a bit	☐ Not at all<br>☐ Somewhat<br>☐ Moderately<br>☐ Quite a bit
5. Participating in social activities outside your home?	☐ Not at all<br>☐ Somewhat<br>☐ Moderately<br>☐ Quite a bit	☐ Not at all<br>☐ Somewhat<br>☐ Moderately<br>☐ Quite a bit	☐ Not at all<br>☐ Somewhat<br>☐ Moderately<br>☐ Quite a bit
6. Emotional health (nervousness, depression, etc)?	☐ Not at all<br>☐ Somewhat<br>☐ Moderately<br>☐ Quite a bit	☐ Not at all<br>☐ Somewhat<br>☐ Moderately<br>☐ Quite a bit	☐ Not at all<br>☐ Somewhat<br>☐ Moderately<br>☐ Quite a bit
7. Feeling frustrated?	☐ Not at all<br>☐ Somewhat<br>☐ Moderately<br>☐ Quite a bit	☐ Not at all<br>☐ Somewhat<br>☐ Moderately<br>☐ Quite a bit	☐ Not at all<br>☐ Somewhat<br>☐ Moderately<br>☐ Quite a bit

Scoring the PFIQ – 7:
All of the items use the following response scale:
0, Not at all; 1, somewhat; 2, moderately; 3, quite a bit

Scales:
Urinary Impact Questionnaire (UIQ-7): 7 items under column heading "Bladder or urine."
Colorectal-Anal Impact Questionnaire (CRAIQ-7): 7 items under column heading "Bowel or rectum."
Pelvic Organ Prolapse Impact Questionnaire (POPIQ-7): 7 items under column heading "Pelvis or vagina."

Scale scores: Obtain the mean value for all of the answered items within the corresponding scale (possible value 0 to 3) and then multiply by (100/3) to obtain the scale score (range 0 to 100). Missing items are dealt with by using the mean from answered items only.

PFIQ-7 Summary Score: Add the scores from the 3 scales together to obtain the summary score (range 0 to 300).
