# Supplementary material for: Translation, reliability, and validity of Amharic versions of the Pelvic Floor Distress Inventory (PFDI-20) and Pelvic Floor Impact Questionnaire (PFIQ-7)
Source: PLoS One. 2022 Nov 17;17(11):e0270434. doi: 10.1371/journal.pone.0270434 (PMC9671332; doi:10.1371/journal.pone.0270434)
Supplement: S1 Table — (DOCX) [file pone.0270434.s004.docx]

S1 Table 1. Floor and ceiling effects of baseline PFDI-20 and PFIQ-7 scores

| Questionnaire | Ceiling, *n (%)* | Floor, *n (%)* |
| --- | --- | --- |
| PFDI-20 | 0 (0) | 4 (2.0) |
| POPDI-6 | 0 (0) | 6 (3.0) |
| CRADI-8 | 0 (0) | 77 (39.1)^a^ |
| UDI-6 | 1 (0.9) | 58 (29.4)^a^ |
| PFIQ-7 | 0 (0) | 8 (4.0) |
| UIQ-7 | 0 (0) | 25 (12.6) |
| CRAIQ-7 | 0 (0) | 53 (26.9)^a^ |
| POPIQ-7 | 0 (0) | 80 (40.6)a |

*PFDI-20* Pelvic Floor Distress Inventory–Short Form 20, *POPDI* Pelvic Organ Prolapse Distress Inventory, *CRADI* Colorectal–Anal Distress Inventory, *UDI* Urinary Distress Inventory, *PFIQ-7* Pelvic Floor Impact Questionnaire-7, *UIQ* Urinary Impact Questionnaire, *CRAIQ* Colorectal–Anal Impact Questionnaire, *POPIQ* Pelvic Organ Prolapse Impact Questionnaire,

*a:* floor effect > 15%
