# Supplementary material for: Translation, reliability, and validity of Amharic versions of the Pelvic Floor Distress Inventory (PFDI-20) and Pelvic Floor Impact Questionnaire (PFIQ-7)
Source: PLoS One. 2022 Nov 17;17(11):e0270434. doi: 10.1371/journal.pone.0270434 (PMC9671332; doi:10.1371/journal.pone.0270434)
Supplement: S2 Table — (DOCX) [file pone.0270434.s005.docx]

S1 Table 2. Correlation between POP-SS and PFDI-20 and PFIQ-7 Scores (Criterion validity)

|  | POP-SS-7 | P-value* |
| --- | --- | --- |
| PFDI-20 | 0.67 | < 0.001 |
| POPDI-6 | 0.71 | < 0.001 |
| CRADI-8 | 0.61 | < 0.001 |
| UDI-6 | 0.63 | < 0.001 |

*POP-SS* Pelvic organ prolapse Symptom Score, *PFDI-20* Pelvic Floor Distress Inventory–Short Form 20, *POPDI* Pelvic Organ Prolapse Distress Inventory, *CRADI* Colorectal–Anal Distress Inventory, *UDI* Urinary Distress Inventory.

*Calculated using Spearman’s rank correlation (SCC) analysis
