# Supplementary material for: Translation, reliability, and validity of Amharic versions of the Pelvic Floor Distress Inventory (PFDI-20) and Pelvic Floor Impact Questionnaire (PFIQ-7)
Source: PLoS One. 2022 Nov 17;17(11):e0270434. doi: 10.1371/journal.pone.0270434 (PMC9671332; doi:10.1371/journal.pone.0270434)
Supplement: S3 Table — (DOCX) [file pone.0270434.s006.docx]

S1 Table 1: Spearman’s correlation coefficient (SCC) between total and subscale scores of PFDI-20, PFIQ-7 and measures of pelvic examination (Pelvic Organ Prolapse Quantification)

|  | PFDI-20 | POP-Q | P-value* |
| --- | --- | --- | --- |
| PFDI-20 | - | 0.69 | < 0.001 |
| POPDI-6 | - | 0.73 | < 0.001 |
| CRADI-8 | - | 0.63 | < 0.001 |
| UDI-6 | - | 0.66 | < 0.001 |
| PFIQ-7 | 0.79 | 0.71 | < 0.001 |
| UIQ-7 | 0.76 | 0.68 | < 0.001 |
| CRAIQ-7 | 0.58 | 0.57 | < 0.001 |
| POPIQ-7 | 0.75 | 0.72 | < 0.001 |

*PFDs* pelvic floor disorders, POP pelvic organ prolapse, *UI* urinary incontinence, *AI* anal incontinence, *PFDI-20* Pelvic Floor Distress Inventory–Short Form 20, *POPDI* Pelvic Organ Prolapse Distress Inventory, *CRADI* Colorectal–Anal Distress Inventory, *UDI* Urinary Distress Inventory, *PFIQ-7* Pelvic Floor Impact Questionnaire-7, *UIQ* Urinary Impact Questionnaire, *CRAIQ* Colorectal–Anal Impact Questionnaire, *POPIQ* Pelvic Organ Prolapse Impact Questionnaire, *POP-Q* Pelvic Organ Prolapse Quantification.

*Calculated using Spearman’s rank correlation (SCC) analysis
